# Evaluation of the improvement of quality of life with Azithromycin in the treatment of eosinophilic nasal polyposis^☆^^☆☆^

**DOI:** 10.1016/j.bjorl.2015.03.018

**Published:** 2015-09-21

**Authors:** Isamara Simas de Oliveira, Paulo Fernando Tormin Borges Crosara, Geovanni Dantas Cassali, Diego Carlos dos Reis, Camilo Brandão de Resende, Flavio Barbosa Nunes, Roberto Eustáquio Santos Guimarães

**Affiliations:** a Department of Surgery and Ophthalmology, Universidade Federal de Minas Gerais (UFMG), Belo Horizonte, MG, Brazil; b Department of Ophthalmology, Otorhinolaryngology and Phonoaudiology, Universidade Federal de Minas Gerais (UFMG), Belo Horizonte, MG, Brazil; c Department of General Pathology, Universidade Federal de Minas Gerais (UFMG), Belo Horizonte, MG, Brazil; d Medicine School, Universidade Federal de Minas Gerais (UFMG), Belo Horizonte, MG, Brazil; e Instituto Tecnológico de Aeronáutica, Universidade Federal de Minas Gerais (UFMG), Belo Horizonte, MG, Brazil

**Keywords:** Eosinophilic nasal polyposis, Azithromycin, SNOT-22, Polipose nasal eosinofílica, Azitromicina, SNOT-22

## Abstract

**Introduction:**

The Sino-Nasal Outcome Test 22 (SNOT-22) is an important tool in assessing the quality of life (QoL) of patients with chronic rhinosinusitis with a validated version in Brazil. The eosinophilic nasal polyposis (ENP) represents most of the cases of nasal polyposis (85–90%) and belongs to the group of chronic rhinosinusitis. It is a chronic inflammatory disease that impacts the QoL of patients, not only causing localized symptoms, but also resulting in a general malaise. The standard treatments (corticosteroids and nasal endoscopic surgery) lead to partial control of symptoms, but relapses are frequent. Macrolide acting as an immunomodulator is a promising tool for more effective control of this disease. Studies are still lacking to assess the real impact on the QoL in patients treated with macrolides.

**Objective:**

To evaluate the improvement of QL, evaluated using SNOT-22, in patients with PNSE treated with immunomodulatory dose azithromycin.

**Methods:**

This is a paired experimental study in patients with ENP. Comparison of pre-treatment and post-treatment with azithromycin was performed. Patients completed the SNOT-22 questionnaire before the start of treatment and returned for evaluation after eight weeks of treatment. Azithromycin was prescribed at a dose of 500 mg, orally, three times a week, for 8 weeks.

**Results:**

SNOT-22 score decreased 20.3 points on average. There was a significant decrease in the SNOT-22 (difference greater than 14 points) in 19 patients (57.6%). There was no significant difference in improvement in SNOT in subgroups with or without asthma/aspirin intolerance.

**Conclusion:**

Azithromycin resulted in significant improvement of QoL, assessed by SNOT-22, in the studied population.

## Resumo

**Introdução:**

O Sino-Nasal Outcome Test 22 (SNOT-22) está entre os principais instrumentos na avaliação da qualidade de vida dos pacientes com rinossinusite crônica, com versão validada no Brasil. A polipose nasossinusal eosinofílica (PNSE) representa a maioria dos casos de polipose nasossinusal (85% a 90%) e pertence ao grande grupo das rinossinusites crônicas. É uma doença inflamatória crônica que impacta sobremaneira a qualidade de vida (QV) dos pacientes, não só pelos sintomas locais, mas também por resultarem em um quadro de mal-estar geral. Os tratamentos padronizados (corticosteroides e cirurgia endoscópica nasal) levam ao controle parcial dos sintomas e as recidivas são frequentes. Os macrolídeos usados como imunomoduladores são uma promissora ferramenta para um controle mais eficaz dessa doença. Ainda faltam estudos para avaliar o real impacto na QV dos pacientes tratados com macrolídeos.

**Objetivo:**

Avaliar a melhora da QV do paciente portador de PNSE tratado com azitromicina em dose imunomoduladora baseando-se questionário SNOT-22.

**Método:**

Trata-se de estudo experimental autopareado em pacientes com PNSE. Foi realizada a comparação dos pacientes pré-tratamento e pós-tratamento com azitromicina. Os pacientes preencheram o questionário SNOT-22 antes do início do tratamento e no retorno após as oito semanas de tratamento. Foi prescrita azitromicina na dose de 500 mg, VO, três vezes por semana, durante 8 semanas.

**Resultados:**

O valor do índice SNOT-22 dos pacientes reduziu 20,3 pontos, em média. Houve diminuição significativa do SNOT-22 (diferença maior que 14 pontos) em 19 pacientes (57,6%). Não houve uma diferença significativa na melhora do SNOT nos subgrupos com ou sem asma/intolerância à aspirina.

**Conclusão:**

A azitromicina resultou em melhora significativa da QV, avaliada pelo questionário SNOT-22, na população estudada.

## Introduction

Quality of life (QoL) is a very important consideration in assessing the severity of nasal disease, clinical effectiveness of treatments used and quality of care of these patients.^1^ Questionnaires for QoL have been frequently used in clinical trials to determine the impact caused by an intervention in certain diseases, or to evaluate the results of health services.^2^

The Sino-Nasal Outcome Test 22 (SNOT-22) is one of the main instruments in assessing QoL of patients with chronic rhinosinusitis, with a Brazilian-validated version.^2^ It is a specific questionnaire for QoL analysis in nasosinusal diseases. With this tool, evaluations of nasal, paranasal, psychological and sleep-related symptoms are carried out.^1^

Eosinophilic nasal polyposis (ENP), is found in approximately 85–90% all cases of sinonasal polyposis.^3^, ^4^, ^5^ This disease belongs to the heterogeneous group of chronic rhinosinusitides (CRS), with involvement of nasal mucosa and paranasal sinuses, being characterized by the presence of edematous formations resulting from prolapse of the lining mucosa.^6^

ENP is the end result of a chronic inflammation of nasal mucosa and paranasal sinuses that greatly impacts QoL of patients, not only by local symptoms (headache, nasal congestion and chronic secretion), but also by resulting in a poor general status.^7^, ^8^

Currently, corticosteroids are considered the main therapeutic option for ENP.^9^ However, the high rates of recurrence of the disease, low response maintenance, persistence of symptoms and side effects of systemic corticosteroids are the main challenges to its treatment.^7^, ^10^, ^11^ Sinus endoscopic surgery is reserved for cases of clinical treatment failure, but even in experienced hands this technique may have recurrence rates ranging from 35 to 50% of cases.^7^, ^12^, ^13^, ^14^ In clinical practice, most patients need both treatments without, however, achieving satisfactory results in controlling the symptoms.^15^

The search for other therapeutic options led to the study of drugs that can act to control the inflammatory process, minimizing those so-feared side effects from chronic use of corticosteroids, in addition to maintaining a prolonged therapeutic response. In this context, macrolides stand out.

Azithromycin is a member of the cyclic-structure macrolide class with 15 elements.^16^ It is the only agent, among macrolides, which does not inhibit the activity of cytochrome P450 (involved in the metabolism of many drugs and in cholesterol and steroid synthesis).^17^ Azithromycin is a well-tolerated agent and, according to the European Position Paper on Rhinosinusitis and Nasal Polyps 2012,^18^ the long-term safety of treatment with macrolides in chronic rhinosinusitis is already established.

The aim of this study was to assess improvement of QoL in patients with ENP treated with an immunomodulatory dose of azithromycin based on the application of SNOT-22.

## Methods

This study was submitted and approved by the Ethics Committee (opinion approval no. 234,835). Patients were informed about the study and its objectives and were requested, after assisted reading and orientation, to sign a post-informed consent form.

The sample consisted of patients with ENP aged 18–60 years and referred by the Brazilian Unified Health System (SUS) to perform surgical treatment in the institution. Such patients, with an established clinical, endoscopic and radiological diagnosis of PNS, had been treated by an optimized standard drug therapy with no appropriate response.

The sample size required for the study was estimated using a significance level of 5% (*α* = 0.05) and statistical power of 80% (*β* = 0.2). To detect a difference of 14 units in the evaluation with SNOT-22 questionnaire and assuming that the standard deviation of the results of this assessment is <28 (*σ*/*ɛ* = 2), the sample size required is 32, based on the formula *n* = (*σ*^2^[*z* (*α*/2) + *z*(*β*)]) * (*σ*/*ɛ*)^2^. Similar studies involved 20 patients.^19^, ^20^

In the screening procedure, the patients underwent a full ENT clinical exam with special attention to the nasal area. The nasal cavity was explored via anterior rhinoscopy and nasofibriolaryngoscopy. PNS was staged according to Sousa et al.^21^ A 3.2-mm diameter flexible nasofibroscope MACHIDA ENT IIIP™ was used. All patients underwent biopsy of nasal polyps to confirm a diagnosis of eosinophilia.

Patients who met the following criteria were selected.

**Inclusion criteria**: Patients with eosinophilic nasal polyposis with eosinophil percentage ≥20% and who, at their clinical and endoscopic examination, showed no evidence of active nasal infection (e.g., purulent discharge in nasal cavity).
**Exclusion criteria**: Patients with non-eosinophilic polyposis such as cystic fibrosis, Kartagener syndrome, antrochoanal polyps and/or eosinophilic nasal polyposis in the presence of infection; patients who used oral or inhaled corticoids or antihistamines or bronchodilators in a period of 30 days preceding the beginning of the study or at any time during the study period; patients with established cardiovascular and/or liver disease and patients with an abnormal electrocardiogram (e.g., QT prolongation).

### General design of the study

This is a self-paired experimental study in patients with ENP. A specific questionnaire was applied before and after treatment with azithromycin, with the aim to compare assessments of QoL.

Further tests for preparation of the surgical procedure were requested: glutamic oxaloacetic transaminase (GOT), glutamic pyruvic transaminase (SGPT) and alkaline phosphatase (AP).

Then, AZI™ (500-mg coated tablets of azithromycin dihydrate, Reg MS: No. 1.3569.0011, EMS S/A, Hortolandia, SP), 1 tablet (500 mg) PO 3×/week (Mondays, Wednesdays and Fridays) for eight weeks was prescribed. In the ninth week, the patient returned to the clinic for further clinical evaluation and for completing a new SNOT-22 questionnaire.

### Variable analyzed

SNOT-22 was translated and adapted to the Portuguese language in 2011.^2^ It consists of 22 questions/symptoms that the patient can sort from zero (no problem) to five (worst problem possible). The patient was advised to answer the questions based on his/her symptoms in the two previous weeks. The sum of the values can range from 0 to 110 points and, the higher this value, the more symptomatic the patient.

With the help of a researcher, the participants completed the SNOT-22 questionnaire before stating the treatment and on their return after eight weeks of therapy.

### Statistical analysis

Paired *t* test was used to compare pre- and post-treatment means. The confidence intervals for the percentages were obtained by the Clopper–Pearson method. The frequency of binary variables in different subgroups was compared using the chi-squared test.

Statistical analyses were performed using the public-domain software Rx64 version 2.15.2, and the conclusions drawn from the results were obtained considering a significance level of 5% and a 95% confidence interval. Data were entered into the database developed on Microsoft Excel™.

## Results

Thirty-three patients completed the study. The patients’ ages ranged from 18 to 69 years, with a mean of 48.84 years, with 21 women (63.6%) and 12 men. Seventeen patients (51.5%) had asthma and/or aspirin intolerance. Only one female patient reported side effects (heartburn/burning) during the use of medication; however, there was no need to discontinue treatment.

At the end of the study, 22 patients reported good symptom control, and chose not to undergo surgical treatment. These patients were kept in clinical follow-up. Eleven patients chose to undergo a surgical procedure, because they did not feel fully satisfied with their post-treatment results.

The normal threshold for the Brazilian version of SNOT-22 is 10 points, and a variation >14 points between SNOT-22 scores of the same patient is considered as significant.^2^ Thus, in the present study, we had one pre-treatment patient and 4 post-treatment patients with a normal SNOT-22 score. Only two patients showed a worse result in SNOT-22 (increases of 1 and 4 points, values considered not significant according to the literature). The SNOT-22 score of all patients decreased by a mean of 20.3 points. Nineteen patients (57.6%) showed a reduction >14 points, considered as significant (*p* < 0.01). It is estimated that, in general, 42–74% of patients show improvement in their post-treatment SNOT-22 evaluation. Table 1 lists an analysis of the results of SNOT-22.

**Table 1:** Post-treatment results of SNOT-22.

Group	*n*	Mean reduction of SNOT-22	Significant reduction (>14)	CI 95% (Clopper–Pearson)
Chose surgery	11	13.6	4 (36.4%)	16.9–69.3%
Did not choose surgery	22	23.7	15 (68.2%)	49.8–86.1%
Total	33	20.3	19 (57.6%)	42.2–74.5%

Non-significant difference (*p* = 0.17) between percentages of groups that chose/did not choose surgery.

The response to the treatment was different in subgroups with/without asthma or aspirin intolerance. A significant difference in reduction of SNOT-22 score between groups with or without asthma/aspirin intolerance was not found (Table 2).

**Table 2:** Subgroup analysis with/without asthma or aspirin intolerance.

Criterion	Asthma/aspirin intolerance		
	Yes (17)	No (16)	*p*-Value
SNOT-22 reduction >14	8 (47.1%)	11 (68.8%)	0.364

## Discussion

The description of QoL is seen as an unique and personal experience, which reflects not only the health status, but also other factors and circumstances of the patient's life.^2^ According to this definition, the comparison between values of SNOT-22 should not be performed among patients, but one can compare the change that occurs before and after any intervention in a given patient.

In patients analyzed in the present study, we observed a significant improvement in QoL assessed by SNOT-22 after treatment with azithromycin for 2 months (8 weeks). These findings corroborate previous studies,^21^, ^22^, ^23^, ^24^ but for the first time a study of a specific and well determined group was carried out. No significant difference between groups with and without asthma/aspirin intolerance was noted.

Interestingly, the improvement in SNOT-22 scores did not affect significantly the patient's decision as to whether or not to undergo surgery, since the decrease in the questionnaire score was not significantly different (*p* = 0.17) for those groups who have chosen or declined surgery.

The SNOT-22 questionnaire has the advantage of combining specific issues of nasosinusal disease with general health issues, which can be evaluated separately or together, before or after treatment.^2^ In line with the authors of the translation, cultural adaptation and validation of the SNOT-22 questionnaire into Portuguese, we choose to standardize the form of application, performing the reading of the questionnaire for our patients, given the difficulties of reading and text comprehension by the population served by our service.

Clinical interpretability is the main challenge for researchers interested in measuring QoL questionnaires, as these tools do not produce intuitively meaningful data, thus making it difficult to interpret the clinical significance of the differences among groups of individuals.^2^

In the last edition of the European Position Paper on Rhinosinusitis and Nasal Polyps 2012,^18^ studies that evaluated the effect of macrolides in nasosinusal polyposis are cited. A moderate effect on reducing the size of the polyps was identified, and this effect has proved more longstanding than that achieved with the use of systemic corticosteroids. However, these studies also failed to assess QoL and clinical benefits for patients.

In the present study, we observed an improvement of QoL measured by the SNOT-22 questionnaire after treatment with azithromycin. Thus, we have demonstrated that the treatment with macrolides can result in real benefit to these patients.

ENP patients, particularly those with asthma and aspirin intolerance, present a difficult-to-control disease, requiring extensive surgery in most cases. Furthermore, in many cases these patients are subjected to several operations throughout their life. In the authors’ opinion, any adjuvant clinical treatment that helps control symptoms and improving QoL of these patients, without causing significant additional side effects, should be considered.

## Conclusion

Treatment with azithromycin 500 mg, 3×/week for 8 weeks, in the population studied showed significant improvement in QoL as assessed by the SNOT-22 questionnaire, with no difference in improvement between groups with and without asthma/aspirin intolerance.

## Conflicts of interest

The authors declare no conflicts of interest.
